# Immunomolecular assay based on selective virion capture by spike antibody and viral nucleic acid amplification for detecting intact SARS-CoV-2 particles

**DOI:** 10.1186/s12951-022-01558-8

**Published:** 2022-09-05

**Authors:** Xiaoli Wu, Junye Liu, Hongpeng Zhang, Hua Zhou, Wen Wang, Yuanyan Ma, Shimei Shen, Xuefei Cai, Ailong Huang, Deqiang Wang

**Affiliations:** 1 The Key Laboratory of Molecular Biology of Infectious Diseases designated by the Chinese Ministry of Education, Department of Infectious Diseases, Institute for Viral Hepatitis, Chongqing Medical University, Yuzhong 400016 Chongqing, China; 2 College of Laboratory Medicine, Chongqing Medical University, Chongqing, 400016 Yuzhong China; 3 Department of Blood Transfusion, Women and Children’s Hospital of Chongqing Medical University, Chongqing, 401147 China; 4 Department of Blood Transfusion, Chongqing Health Center for Women and Children, Chongqing, 401147 China; 5 Department of Clinical Laboratory, The Second Affiliated Hospital of Chongqing Medical University, Chongqing, 400010 Yuzhong China

**Keywords:** COVID-19, Complete virions, Spike, Infection risk, Immunomolecular detection

## Abstract

**Background:**

Effective therapeutics and vaccines for coronavirus disease 2019 (COVID-19) are currently lacking because of the mutation and immune escape of severe acute respiratory syndrome coronavirus 2 (SARS-CoV-2). Based on the propagation characteristics of SARS-CoV-2, rapid and accurate detection of complete virions from clinical samples and the environment is critical for assessing infection risk and containing further COVID-19 outbreaks. However, currently applicable methods cannot achieve large-scale clinical application due to factors such as the high viral load, cumbersome virus isolation steps, demanding environmental conditions, and long experimental periods. In this study, we developed an immuno molecular detection method combining capture of the viral spike glycoprotein with monoclonal antibodies and nucleic acid amplification via quantitative reverse transcription PCR to rapidly and accurately detect complete virions.

**Results:**

After constructing a novel pseudovirus, screening for specific antibodies, and optimizing the detection parameters, the assay achieved a limit of detection of 9 × 10^2^ transduction units/mL of viral titer with high confidence (~ 95%) and excellent stability against human serum and common virus/pseudovirus. The coefficients of variation were 1.0 ~ 2.0% for intra-assay and inter-assay analyses, respectively. Compared with reverse transcription-PCR, the immunomolecular method more accurately quantified complete virions. SARS-CoV-2/pseudovirus was more stable on plastic and paper compared with aluminum and copper in the detection of SARS-CoV-2 pseudovirus under different conditions. Complete virions were detected up to 96 h after they were applied to these surfaces (except for copper), although the titer of the virions was greatly reduced.

**Conclusion:**

Convenient, inexpensive, and accurate complete virus detection can be applied to many fields, including monitoring the infectivity of convalescent and post-discharge patients and assessing high-risk environments (isolation rooms, operating rooms, patient living environments, and cold chain logistics). This method can also be used to detect intact virions, including Hepatitis B and C viruses, human immunodeficiency virus, influenza, and the partial pulmonary virus, which may further improve the accuracy of diagnoses and facilitate individualized and precise treatments.

**Graphical Abstract:**

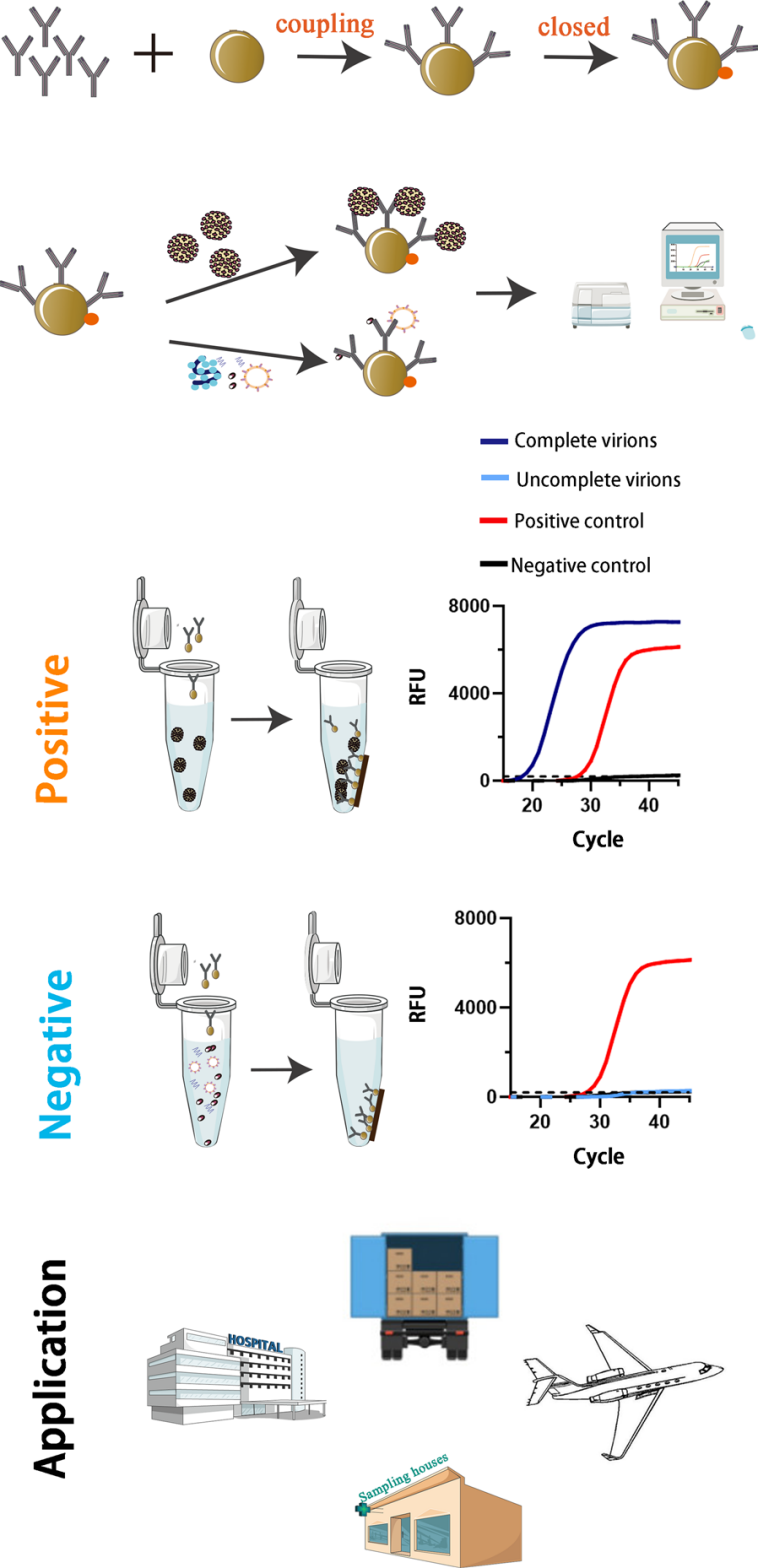

**Supplementary Information:**

The online version contains supplementary material available at 10.1186/s12951-022-01558-8.

## Background

Coronavirus disease 2019 (COVID-19), caused by the novel coronavirus severe acute respiratory syndrome coronavirus 2 (SARS-CoV-2), has developed into a serious global public health threat [1, 2]. Despite extensive financial investments into the diagnosis, treatment, and prevention of COVID-19, the pandemic remains ongoing because of the high infectivity, pathogenicity, mutation and immune escape of SARS-CoV-2 [3–6].

The novel β-coronavirus, SARS-CoV-2, contains a single-strand positive-sense RNA genome of 30 kilobases containing five major open reading frames (ORFs): replicase complex (ORF1ab), spike (S), envelope (E), membrane (M), and nucleocapsid (N), and is approximately 80–120 nm in diameter [7–9]. SARS-CoV-2 enters and infects the host cell via the S protein on the outer viral membrane by interacting with the host receptor human angiotensin-converting enzyme 2 (hACE2) [10–12]. The positive single-stranded RNA is released from the virus and moves into the cell nucleus, where nonstructural proteins, viral polymerases, RNA, and viral structural proteins are sequentially synthesized. These components are then packaged into new complete virion particles. The progeny virus is wrapped in a vesicle in the cytoplasm and released from the cell through exocytosis to begin the next viral cycle of infection [13]. Analogously, the nucleocapsid protein can form condensates with viral genomic RNA which are secreted extracellularly as “subviral particles” and is a common occurrence in the life cycle of human viruses [14–16]. For example, the core protein and viral DNA of Hepatitis B virus (HBV) can assemble as capsid particles that are secreted, and the capsid protein (CA) of human immunodeficiency virus (HIV) and core protein of Hepatitis C virus can coat viral RNA and emit them as sub-virus particles [17–20]. Based on the life cycle of SARS-CoV-2, intact virus particles clearly represent the only elements that sustain infection, whereas subviral particles cannot sustain the viral life cycle consistently enough to persistently infect the host.

According to previous studies, human-to-human transmission via respiratory droplets and close contact is the main transmission route of SARS-CoV-2 [21, 22]. However, some cold chain practitioners have been infected via contact with contaminated cold chain goods globally; therefore, contact with objects contaminated by SARS-CoV-2 may also cause infection [23–25]. Recent studies reported that positive results for COVID-19 were detected in blood samples of convalescent patients and different environmental conditions, including cold-chain logistics, hospital rooms, and airtight cabins [26–31]. Whether positive signals indicate that the patient or environment is at a high risk of infection, as well as the influence of subviral particles and free RNA fragments, requires further analysis.

Several methods have been developed to ensure the timely and effective detection of SARS-CoV-2 infection, including pathology-based chest computed tomography, protein-based viral antigens, and antibody detection and nucleic acid-based RNA detection [32–41]. These assays are important for diagnosing coronavirus infections and forming clinical treatment prognoses [42]. However, some “subviral particles” are either missing both the genome and capsid, or missing just the genome, resulting in false-positive detection signals that do not truly reflect the viral replication and infection abilities. For example, HBV subviral particles composed of only viral surface proteins are present in the blood of infected individuals at up to a 100,000-fold excess compared to complete virions (at 10^14^/mL). Empty (genome-free) virions, which contain the surface proteins enclosing the viral capsid but no genome, exhibit 100-fold higher levels over complete virions in the blood of infected individuals (10^11^/mL) [43–46]. Therefore, detecting intact SARS-CoV-2 particles in body fluid (including blood, saliva, etc.) and the environment is critical for assessing infection risk and containing further outbreaks of COVID-19.

Using pseudoviruses enables research of highly pathogenic viruses without the requirement for highly secure biosafety facilities, which has greatly benefited research on COVID-19 [47–50]. However, the previously reported SARS-CoV-2-related recombinant virus either simulated the functional structure of S and N protein or incorporated fragments of its nucleic acids, and failed to simulate the functional structure of complete SARS-CoV-2 particles. Here, we constructed a SARS-CoV-2 pseudovirus system to simulate the functional structure of SARS-CoV-2 and obtained monoclonal antibodies with high affinity through screening of 11 strains of specific antibodies directed to S proteins. An immunomolecular assay was developed by combining immunocapturing SARS-CoV-2 spike protein and nucleic acid amplification via quantitative reverse transcription polymerase chain reaction (RT-qPCR) for highly sensitive and selective detection of intact SARS-CoV-2 particles.

## Results

### Construction of pseudo-SARS-CoV-2 virus and screening of specific antibodies directed to spike glycoproteins

**Fig. 1:**
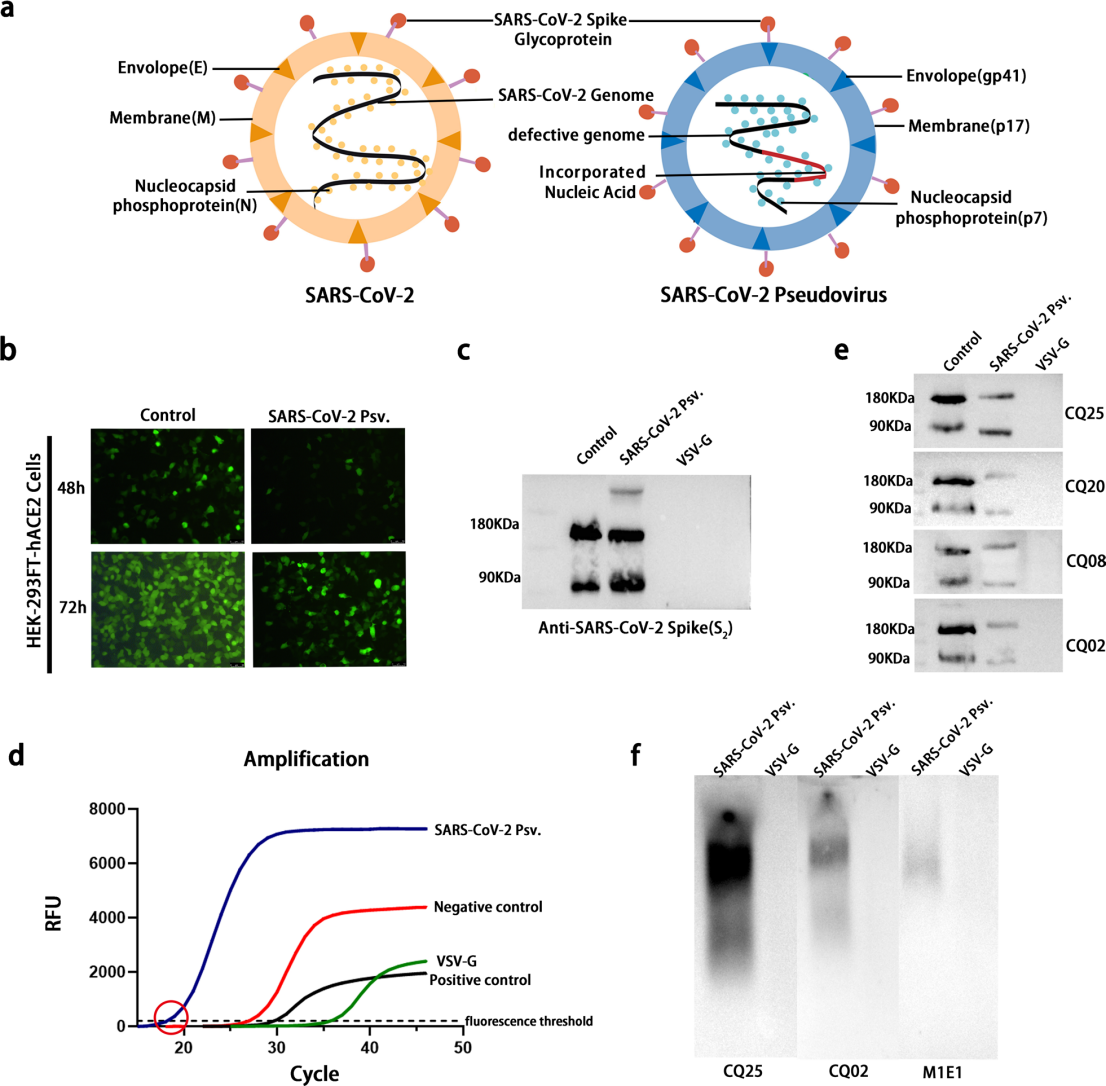
Construction of pseudo-SARS-CoV-2 virus and screening of specific antibodies directed to spike proteins. **a** Structures of SARS-CoV-2 and the SARS-CoV-2 pseudovirus. **b** Infectivity of SARS-CoV-2 pseudovirus. HEK-293FT-hACE2 cells transduced with VSV pseudovirus encoding GFP were used as a positive control. **c** Detection of SARS-CoV-2 S protein incorporated in lentivirus via western blotting. The wild-type spike glycoprotein overexpressed in 293 cells and VSV pseudovirus without spike glycoprotein were used as the positive control and the negative control, respectively. **d** Detection of target sequence synthesized and cloned in SARS-CoV-2 lentivirus via RT-qPCR using a 2019-nCoV nucleic acid detection kit (Sansure Bio, China). The VSV pseudotype virus was prepared using the same procedure without the target sequence taken as the negative sample. **e** Identification of potential antibodies binding to the polypeptide fragment of spike glycoproteins via western blot with the lysate of SARS-CoV-2 pseudovirus after heating for 10 min at 100 °C. The SARS-CoV-2 S protein expressed via 293T cells transfected with vector-encoded wild-type SARS-CoV-2 S glycoprotein used as a control. **f**. Identification of potential antibodies binding to the active Spike glycoproteins of SARS-CoV-2 through the particle gel with pseudovirus resuspension. The VSV pseudotype virus was prepared suing the same procedure and used as a negative control

To simulate the functional structure of intact SARS-Cov-2 particles, we constructed a SARS-CoV-2 pseudovirus expressing the spike glycoproteins outside the envelope and incorporating four viral uncompleted genes of SARS-CoV-2 (ORF1ab, ORFab, N, and M) into the lentivirus encoding green fluorescence protein (GFP) (Fig. 1a). A lentivirus-transformed plasmid, pLV-SARS-CoV-2-F1abFabNE-GFP, and an envelope plasmid, pCMV3-2019-nCoV-Spike (S1 + S2), were constructed first (Additional file 1: Fig. S1). After generating SARS-CoV-2 pseudoviruses through co-transfection, we tested their infectivity by transducing HEK-293FT-hACE2 cells (Additional file 1: Fig. S2). Abundant GFP fluorescence was observed via fluorescence microscopy at 48 and 72 h, suggesting successful construction and transduction of the pseudovirus (Fig. 1b). The efficiency of incorporating the SARS-CoV-2 S protein and targeted sequence into the lentiviral cells was evaluated using monoclonal mouse anti-S (S2 domain) via western blotting and qRT-PCR targeted to the F1ab gene, respectively (Fig. 1c, d). The wild-type spike glycoprotein overexpressed in 293 cells and VSV pseudovirus without spike glycoprotein were used as the positive and negative controls, respectively (Fig. 1c). Consistent with the positive control, the SARS-CoV-2 pseudovirus also had specific bands at 190 and 80 kDa corresponded to the monomer S protein (S1 + S2) and S2 domains, respectively, indicating that the spike glycoprotein was successfully incorporated into the pseudovirus (Fig. 1c, lane 3). qRT-PCR targeted to the F1ab gene was used to verify whether the targeted sequence was successfully inserted into the SARS-CoV-2 pseudovirus and VSV pseudovirus without the target sequence being taken as the negative sample. The fluorescence intensity of SARS-CoV-2 pseudovirus successively progressed through the stage of fluorescence background signal, fluorescence signal index amplification and the plateau in real-time fluorescence quantitative amplification and the cycles exceeding the set fluorescence threshold was smaller than the negative control which was a positive amplification signal (Fig. 1d). The intact SARS-CoV-2 pattern was successfully constructed by combining the western blotting and qRT-PCR with the results of cell transduction.

Based on the SARS-CoV-2 pseudovirus, 11 monoclonal antibodies (CQ2, CQ20, CQ25, CQ8, CQ12, CQ001, CQ100, CQ040, CQ042, CQ023, and M1E1) directed to the spike glycoproteins of SARS-CoV-2 were prepared and screened according to their affinity using western blotting and particle gel assay [51]. After lysis and denaturation electrophoresis of SARS-CoV-2 pseudovirus, the four antibodies, including CQ25, CQ20, CQ08 and CQ2, reacted with the polypeptide fragment of spike glycoproteins (Fig. 1e). Whereas, the active spike glycoproteins of SARS-CoV-2 influenced by the natural shape and charge only were detected with the CQ2, CQ25, and M1E1 antibodies (Fig. 1f). Among these 11 candidates, the CQ25 antibodies demonstrated excellent affinity and specificity for S protein.

### Immunomolecular detection platform for SARS-CoV-2/pseudovirus particles

**Fig. 2:**
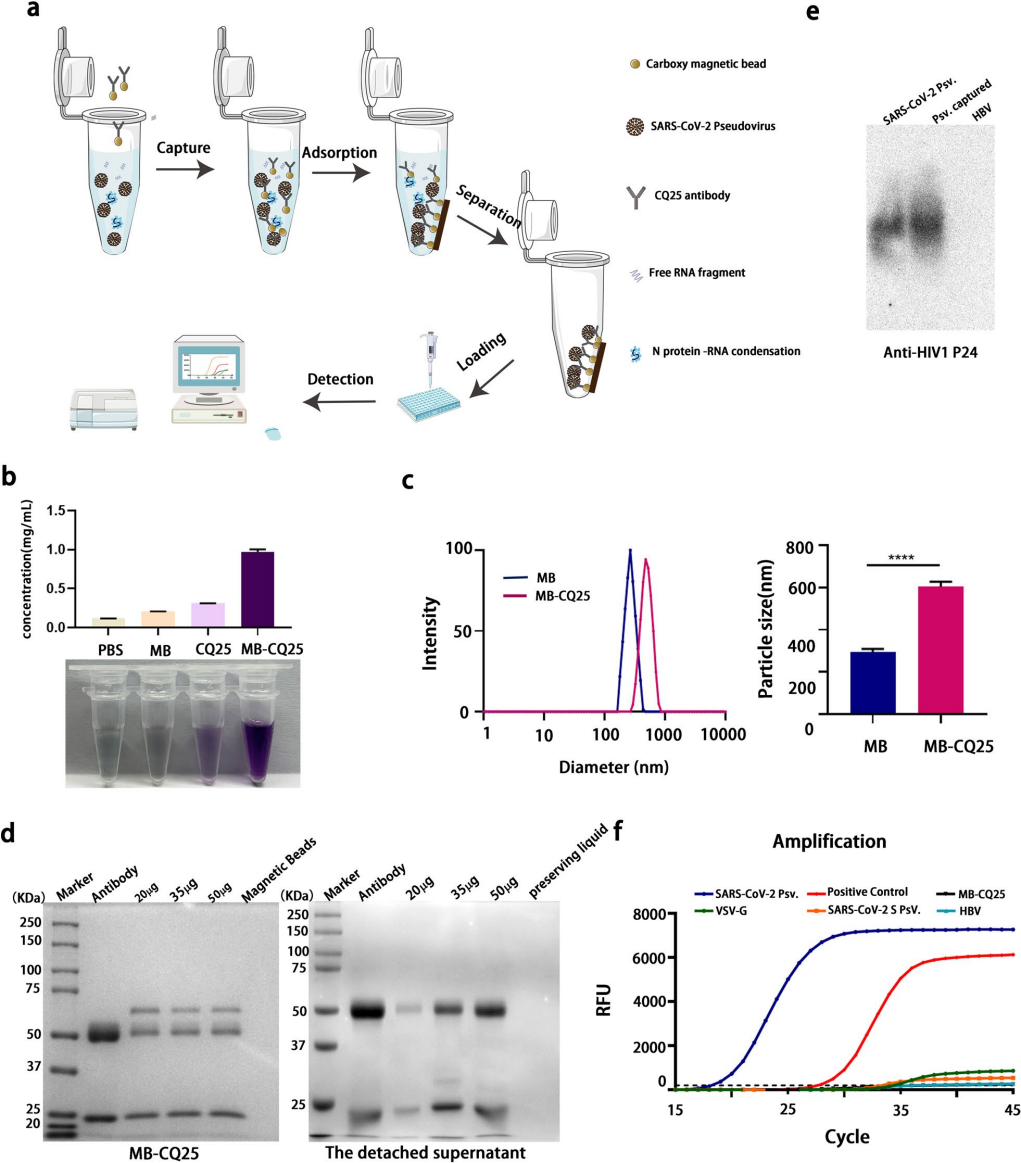
Establishment of immunocapture‐based SARS-CoV-2 pseudovirus detection platform. **a** Flow diagram of immunocapture-based SARS-CoV-2 pseudovirus detection platform. **b** Protein concentration of carboxyl magnetic bead-antibody complexes following conjugation evaluated in a BCA assay. **c**. Particle size analysis of carboxy magnetic beads (MB) and magnetic beads coupled with a CQ25 antibody (MB-CQ25) (****, P < 0.0001). **d** Optimization of the antibody required for conjugation with beads. Carboxyl magnetic beads coupled with a CQ25 antibody and corresponding supernatant were detected using SDS-PAGE. **e** SARS-CoV-2 pseudovirus captured via carboxyl magnetic beads coupled with a CQ25 antibody was detected using a particle gel assay with anti-HIV1 P24 antibodies. **f** The real-time fluorescence quantitative amplification targeted to the F1ab. Multiple viruses captured via CQ25 antibody-coupled carboxyl magnetic beads were detected using qRT-PCR with a 2019-nCoV nucleic acid detection kit (Sansure Bio)

After preparing the SARS-CoV-2 pseudovirus and high-affinity antibodies, an immunomolecular detection platform for intact SARS-CoV-2/pseudovirus was designed. First, the carboxyl groups on the magnetic beads were activated with EDC(1-(3-Dimethylaminopropyl)-3-ethylcarbodiimide Hydro) and sulfo-NHS or NHS (N-hydroxysuccinimide) by forming dry-stable Amine-reactive sulfo-NHS Ester. The activated carboxyl group on the magnetic bead formed an amide bond with the free amino group of the anti-spike antibody, thus, successfully coupling the antibody with the magnetic bead [52]. Second, after co-incubation for 30–45 min at 20–25 °C, antibodies-beads complexes specifically captured the SARS-CoV-2/pseudovirus particles. Third, following separation of the supernatant using a magnetic separator, the SARS-CoV-2/pseudovirus particles were enriched and separated from other “subviral particles” without S glycoprotein and free RNA fragments. Finally, nucleic acid-based detection via qRT-PCR excluded the influence of empty (genome-free) virions to further ensure that the detection platform only targeted intact SARS-CoV-2/pseudovirus particles (Fig. 2a).

To establish an immunomolecular detection platform for intact SARS-CoV-2 particles, carboxyl magnetic beads and anti-Spike antibody (CQ25) were coupled. The effect of conjugation was evaluated by measuring the protein concentration of the complexes following conjugation using a BCA assay (Fig. 2b) and particle size analysis (Fig. 2c). The CQ25 alone group was used as a positive control for the success of conjugation in Fig. 2b. Compared with pre-conjugated antibody (the CQ25 alone group), MB-CQ25, on which the CQ25 antibody was enriched, showed much higher absorption intensities (approximately tenfold) at 562 nm. In addition, compared with pre-conjugated magnetic beads (MB), MB-CQ25 demonstrated much larger average effective diameters (approximately twofold) (Fig. 2c). Both of these results proved that the magnetic beads were successfully coupled with the antibody. Gradient concentrations of CQ25 antibody (20 mg, 35 mg and 50 mg) were used to ensure that the beads were completely saturated and to exclude non-specific adsorption of nucleic acids. Pre-conjugated antibody (lane 1) and carboxyl magnetic beads (lane 5) were used as a positive control and negative control in Fig. 2d, respectively. The complexes and corresponding supernatant were subjected to sodium dodecyl sulfate–polyacrylamide gel electrophoresis (SDS-PAGE) followed by Coomassie bright blue staining, which confirmed the successful coupling and optimization of the coupling parameters (Fig. 2d). Next, we verified whether the SARS-CoV-2 pseudovirus could be captured by anti-S antibody-magnetic beads complexes (MB-CQ25) at the protein and nucleic acid levels. As HIV1 P24 is the most abundant marker protein in the lentivirus capsid, we identified the viral capsid proteins of captured virions in a particle gel assay with the monoclonal mouse anti-HIV 1 p24 antibody. As shown in Fig. 2e, specific bands were observed in the complexes following capture. Importantly, compared with the negative control samples, including the SARS-CoV-2 S pseudovirus, VSV-G pseudovirus, and HBV, only our SARS-CoV-2 pseudovirus generated a positive signal via nucleic acid-based detection following capture (Fig. 2f). These results indicated that the immunomolecular detection platform specifically detected intact SARS-CoV-2/pseudovirus particles.

### Intact viral particles as the sole target of immunomolecular assay

**Fig. 3:**
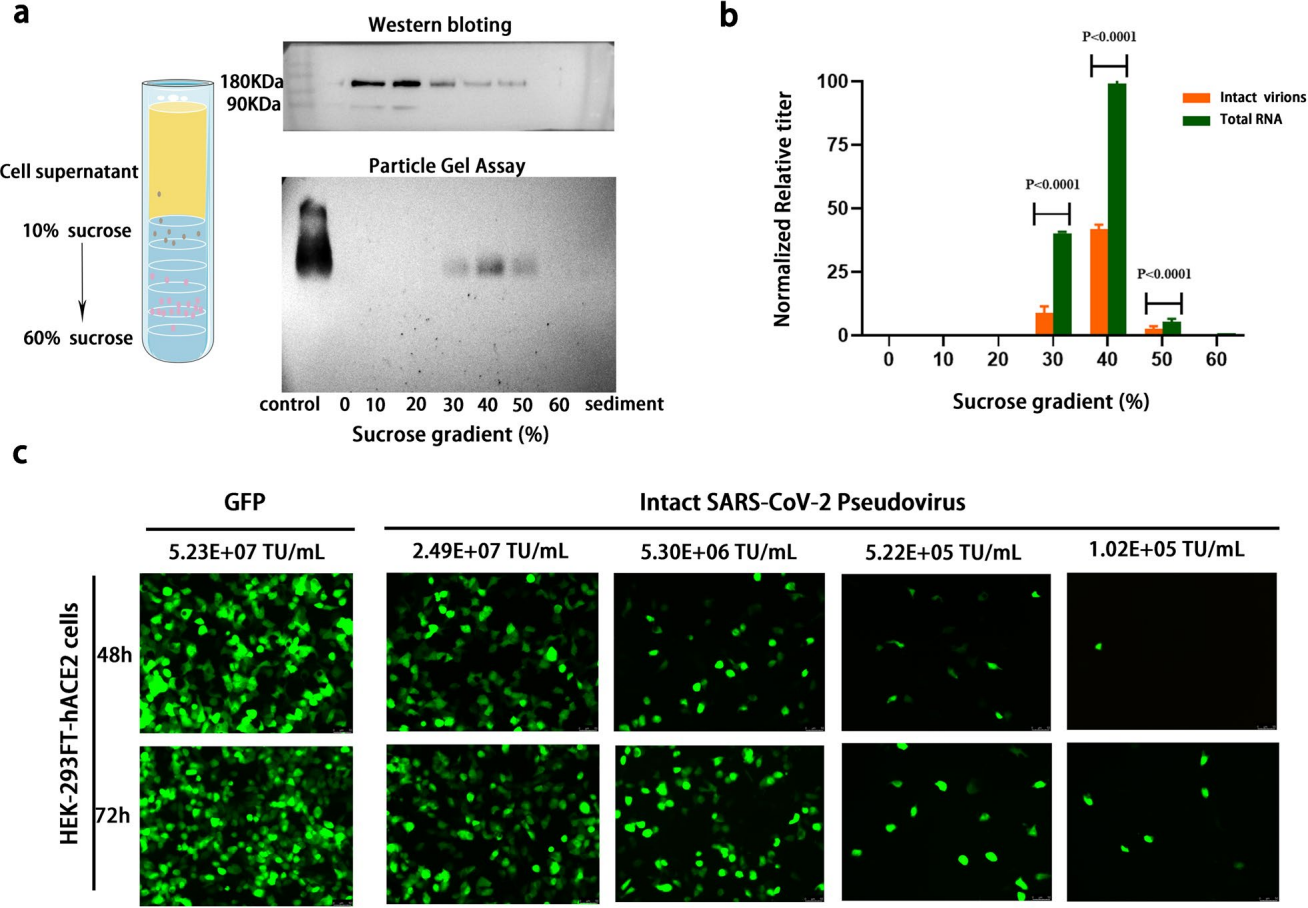
Detection target of immune molecular method is only intact virus particles. **a** Levels of spike glycoprotein of SARS-CoV-2 pseudovirus in each gradient fraction analyzed by the western blotting and particle gel assay using anti-spike (S2) antibodies, respectively. **b** Levels of F1ab gene of SARS-CoV-2 pseudovirus in each gradient fraction analyzed via qRT-PCR and immunomolecular assay, respectively. **c** Infectious activity corresponding to different titers of complete virions

To verify the detection target of the immunomolecular assay, the HEK-293FT cell supernatant containing virus particles with different structures and genetic components after being co-transfected with the HIV-1 lentivirus vector for 72 h was collected and separated by sucrose density gradient centrifugation [46]. Western blotting and particle gel assay were used to identify the content of spike glycoprotein and intact virus particles in the supernatant, respectively. As shown in Fig. 3a, the S glycoprotein peaked in the 10–20% sucrose gradient, whereas the pseudovirus particles settled at the 40% sucrose gradient, suggesting the presence of abundant free S proteins (large excess over complete virions) in the cell supernatant. Additionally, qRT-PCR and immunomolecular assays were used to determine the RNA levels of total and intact virions in different density gradients (Fig. 3b). The data indicated that intact pseudovirus particles were enriched in the 40% sucrose gradient fraction consisting with Fig. 3a and the virogene level was significantly higher than the intact virion (P < 0.0001), suggesting that some subviral particles (without spike proteins) were produced during virus packaging; these results are consistent with previous reports on HBV [44, 45]. Using different titers of intact virions to transduce 293FT-HEK-hACE2 cells, we determined that the titer of intact virions detected in the immunomolecular assay was positively correlated with infectivity (Fig. 3c). These results indicated that the detection target of the immunomolecular assay is intact virus particles. In addition, detections based on antigen and nucleic acid do not accurately reflect infection status, detection based on intact virus particles is necessary and urgent.

### Validation of immunomolecular assay for intact SARS-CoV-2 particles

**Fig. 4:**
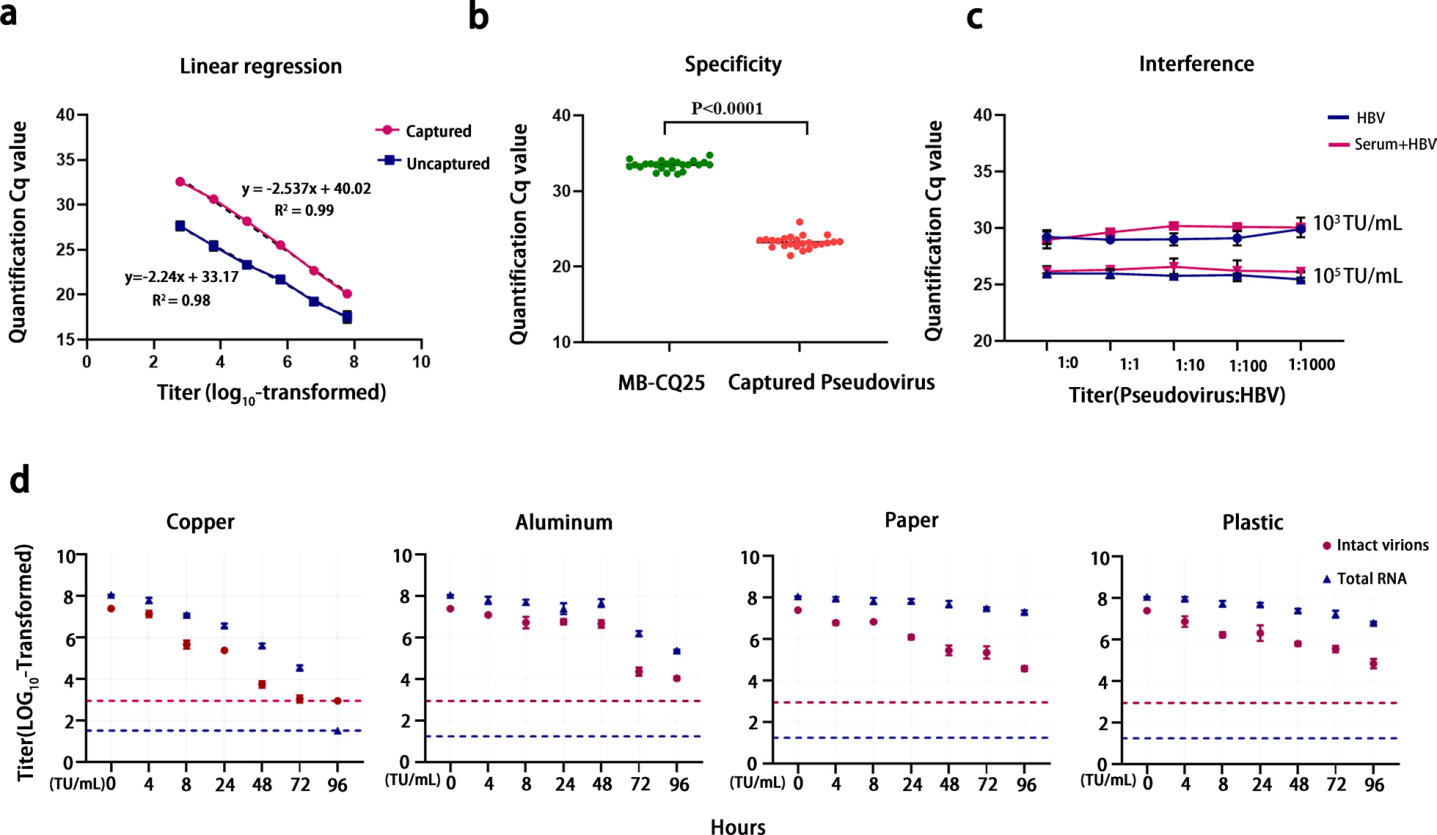
Validation of immunomolecular assay for intact SARS-CoV-2 particles.** a** Linear regression for the assay. When the SARS-CoV-2 pseudovirus titer ranged between 6 × 10^2^ and 6 × 10^7^ TU/mL, a linear relationship occurred between the quantification Cq value and its titer (log-transformed) in the immunomolecular assay. y = − 2.537x + 40.02, R^2^ = 0.99. **b** Specificity of the assay. Twenty-four of the carboxyl magnetic beads with CQ25 antibody complexes (MB-CQ25) and 24 captured pseudovirus samples were used to evaluate the assay specificity. ****P < 0.0001. **c** Interference from HBV supernatant from AD38 cells and patient serum with different titers of HBV. **d** SARS-CoV-2 pseudovirus stability under different conditions. Viruses were applied to copper, aluminum, paper, and plastic maintained at 21–23 °C and 40% relative humidity over seven days. The titer of viable virus was expressed as log_10_-tranformed. Plots show the means and standard errors (I bars) of three replicates

After optimizing the detection conditions, ten-fold gradient dilutions were prepared and analyzed using fluorescence qRT-PCR in parallel to determine the linear range (Additional file 1: Fig. S3). When the titer ranged between 6 × 10^2^ and 6 × 10^7^ transduction units (TU)/mL, there was a linear relationship between the Cq value and its titer (log_10-_transformed) in the immunomolecular assay (R^2^ = 0.99) (Fig. 4a). As shown in Fig. 4b, there were significant differences between the negative samples with the 24 carboxy magnetic beads coupled with CQ25 antibodies (MB) and positive samples containing 24 instances of captured pseudovirus (P < 0.0001). Using a mean titer of the negative samples of + 1.96 standard deviation, the limit of detection was 900 TU/mL. The results of fluorescence quantitative PCR were negative despite the Cq value being measured when the pseudovirus titer was lower than 900 TU/mL.

Human serum contains a large quantity of albumin and various antibodies, which may affect assay stability. Additionally, if the patient carries other non-specific viruses, the specificity and stability of the method may be affected. To evaluate these factors, the HBV supernatant collected from AD38 cells and patient serum with different titers of HBV were used to verify the anti-interference ability of this immunomolecular assay. As shown in Fig. 4c, for the low-titer group, adding HBV and patient serum with different titers of HBV had a negligible effect on the quantitative qualitative detection rate of intact SARS-CoV-2 pseudovirus particles, with coefficients of variation of 1.5% and 2.0% for intra-assay and inter-assay analyses, respectively; those of the high-titer group were 1.4% and 1.0%. Thus, this method is significantly stable against non-specific viruses and human serum. We further examined the stability of the SARS-CoV-2 pseudovirus over time on various surfaces, including copper, aluminum, paper, and plastic, to mimic different environmental samples (Fig. 4d). SARS-CoV-2 was more stable on plastic, aluminum, and paper than on copper, and viable virus was detected up to 96 h after application to these surfaces; however, the virion titers were greatly reduced (from 10^7.38^ to 10^4.05^ TU/mL on aluminum, from 10^7.38^ to 10^4.58^ TU/mL on paper, and from 10^7.38^ to 10^4.84^ TU/mL on plastic). Notably, although the total RNA produced a positive detection signal, live virus was not detected on the copper surface at 72 h. Moreover, the gap in the titer between the total RNA and intact virions increased over time under different conditions. These results suggest that RNA fragments exhibit different stabilities under different conditions following complete virions degradation, supporting the theory that RNA levels alone should not be used to assess infection risk.

## Discussion

Based on the transmission characteristics of SARS-CoV-2, rapidly screening for positive cases and contaminated environments is critical to control the spread of COVID-19. Traditional detection methods based on nucleic acid fragments or protein are affected by “subviral particles,” such as free RNA fragments, free antigens, nucleocapsid protein-RNA condensates, and empty virions, and thus have failed to provide an accurate basis for patient medication and environmental assessment. Previously, a platform coupled to Raman spectroscopy was used to capture viruses based on their size; however, the nonspecific and harsh experimental conditions prevented application of this approach in clinical laboratories [53]. The median tissue culture infective dose was also used to quantify virus titers and assess infectivity. However, the high viral load, cumbersome virus isolation steps, demanding environmental conditions, and long experimental periods made it difficult to apply this method in clinical laboratories [54–56]. As free antigens in the supernatant are present in much greater excess over complete virions, detection of viral particles based on viral antigens cannot completely reflect the infection risk of patients and the environment [37, 39]. Here, we developed an immunomolecular assay for detecting intact SARS-CoV-2 particles. This method achieved excellent stability and specificity against other common human virus/pseudovirus, and excellent accuracy and convenience for detecting intact SARS-CoV-2 particles in environmental samples. Compared to previously reported pseudoviruses, our designed pseudo-SARS-CoV-2 virus better simulated intact SARS-CoV-2 particles at the nucleic acid and protein levels. Four fragments of viral RNA sequences, ORF1ab (15415–15540), ORFab (12750–13491), N gene (28750–29150), and E gene (26360–26381), wrapped in the genome of the pseudovirus were detected using all nucleic acid detection reagents recommended by the World Health Organization and can be sold on the market without redesigning the primers used in qRT-PCR. In addition, compared with previously reported SARS-CoV-2 biomimetic virus-like particles with ∼16% variation, our novel pseudovirus shows much higher stability and may serve as a quantitative diagnostic tool for SARS-CoV-2 (Additional file 1: Fig. S4) [57].

This assay can be used in a variety of scenarios including monitoring the infectivity of convalescent and post-discharge patients and assessing the risk of infection in high-risk environments including virus detection sampling houses, international flight cabins, and international cold-chain logistics bases. For research and applications, affinity antibodies and carboxyl magnetic beads can be optimized to improve the detection sensitivity. Moreover, isothermal nucleic acid amplification methods may be combined with this detection method to further simplify the experimental process and reduce professional dependence on experimental equipment, enabling applications of this method in clinical practice. This immunomolecular assay can also be used to detect other intact virus particles such as HBV, Hepatitis C virus, HIV, influenza, and the partial pulmonary virus, which may improve the accuracy of diagnoses of these infectious diseases and facilitate individualized and precise treatments. However, the potential applications for detecting these intact virus particles requires further experimental studies.

## Conclusion

We prepared a pseudovirus that fully simulates the SARS-CoV-2 virus in a safe and convenient manner. After screening monoclonal antibodies with high affinity for S proteins, we developed an immunomolecular assay combined immunocaptured of SARS-CoV-2 S protein and nucleic acid amplification via qRT-PCR for highly sensitive and selective detection of intact SARS-CoV-2 particles. This method enables accurate assessment of the contagiousness of infected patients and/or potential infection hazard in high-risk environments.

## Methods

### Production and titration of pseudoviruses

A plasmid containing partial sequences of the SARS-CoV-2 ORF1ab gene, N gene, E gene, and GFP reporter was constructed and named as PLV-SARS-CoV-2-F1abMEN-GFP. Pseudoviruses were produced and titrated using methods similar to the Rift Valley Fever pseudovirus, as previously described [58]. Briefly, HEK-293FT cells were transfected with the plasmids pCMV3-2019-nCoV-Spike(S1 + S2), pLV-SARS-CoV-2-F1abFabME-GFP, and pMD2.G (Sino Biological, Inc. Beijing, China) using Lipofectamine 8000 (C0533, Beyotime Biotechnology, Shanghai, China). The virus supernatant was collected and mixed at 48 and 72 h after transfection and centrifuged at 4 °C at 4000 × *g* for 10 min to remove cell debris. The culture supernatant was placed on a 20% sucrose solution and centrifuged at 25,000 rpm (112,000 × *g*) for 15 h at 4 °C in a Beckman SW28 rotor (Beckman Coulter, Brea, CA, USA). For the HIV 1 pseudovirus system, the titer of PsVs was quantified using an HIV-1 Gag p24 DuoSet ELISA kit (KIT11695, Sino Biological Inc. Beijing, China) according to the manufacturer’s instructions.

After transfection with lentivirus overexpressing hACE2 or an empty vector at 48 h, 1 × 10^5^ HEK-293FT cells in a 96-well plate were transduced with the SARS-CoV-2 pseudovirus and control pseudovirus encoding GFP at a multiplicity of infection of 2 vp/cell for 24 h at 37 °C. Infection was visualized using a fluorescence microscope at 48 and 72 h.

### Conjugation of carboxyl magnetic beads with an antibody

The activating reagent, antibody and blocking reagent used in the conjugation procedure were prepared with 25 mM MES buffer (PH6.0). Next, 3.3 mg carboxyl magnetic beads (1 mm, 20 mg/mL) were cleaned with 200 mL MES buffer twice, then 100 µɅ NHS and 100 µɅ EDC solution (25 g/L) were successively added, and the mixture was shaken vigorously for 30 s and then mixed at 25 °C for 30 min. After the activating reagent was discarded, 66 mg of antibody (diluted to 0.6 mg/mL) was added and mixed at 4 °C for 4 h. Subsequently, the carboxyl reactive ester on the beads was sealed with 1% bovine serum albumin solution at room temperature for 30 min, finally the magnetic beads were re-suspended with 120 mL preservation solution and stored at 4 °C. SDS-PAGE and Coomassie blue staining were performed to evaluate the coupling effect using coupled magnetic beads and separate the supernatants.

### qRT-PCR

SARS-CoV-2 RNA levels were detected using qRT-PCR on a CFX96 system (Bio-Rad, Hercules, CA, USA) using a 2019-nCoV nucleic acid detection kit (fluorometric real-time PCR) (001, Sansure Bio, Hunan, China). Each sample was evaluated in triplicate, and two no-template control wells were included to confirm that there was no contamination.

### Western blotting

The samples were mixed with 6 × SDS sample buffer, boiled for 10 min at 95 °C, and subjected to SDS-PAGE and immunoblotting. The pseudovirus was detected using mouse/human anti-S/membrane antibodies (CQ2, CQ20, CQ25, CQ8, CQ12, CQ001, CQ100, CQ040, CQ042, CQ023, and M1E1; a gift from the Laboratory of Molecular Biology of Infectious Diseases, Chongqing Medical University, Chongqing, China) at a 1:1000 dilution. Goat anti-mouse IgG (No. SA00003-1, Proteintech, Rosemont, IL, USA) was used at a 1:4000 dilution as the secondary antibody.

### Particle gel assay

Denatured samples were resolved on a 1% agarose gel in 1 × TAE buffer at 70 V for 2 h. Particles in the gel were transferred to a positively charged microporous nylon membrane in 1 × TBE buffer for 12 h based on the Siphon principle [51]. After transfer, the membrane was blocked in 5% bovine serum albumin for 30 min and incubated in an anti-HIV-1 P24 antibody solution or anti-S antibody solution for 12 h at 4 °C. The membranes were exposed following washing with 1 × Tris-buffered saline containing 1‰ Tween 20.

### Particle size analysis

The effective particle size of the carboxyl magnetic beads (MB) and magnetic beads-CQ25 antibody complex (MB-CQ25) were characterized using a NanoBrook 90PLUS PALS particle size analyzer (Brookhaven Instruments Corporation, Holtsville, NY, USA). The sample was added to pure water (1‰ Triton-100) and mixed to ensure even dispersion in the medium. If necessary, the sample was sonicated for 15 min. The particle size was determined after setting the parameters according to the instrument manual. Three complex holes were placed each time, and this process was repeated twice. The data were analyzed and plotted using GraphPad Prism 8 software (GraphPad Inc., San Diego, CA, USA).

### Sucrose density gradient ultracentrifugation

Sucrose density gradient centrifugation was performed as previously described [46]. Briefly, discontinuous sucrose density gradients (10%, 20%, 30%, 40%, 50%, and 60%) were prepared in PBS. The culture supernatant was laid on the linear sucrose gradient and centrifuged at 25,000 rpm (112,000 × *g*) for 15 h at 4 °C in a SW28 rotor (Beckman Coulter).

### Statistical analyses

Linear regression, descriptive statistics, repeated measures analysis of variance, and two groups of unpaired *t*-tests were performed using the statistical software package SPSS version 21.0 for Windows (SPSS Inc., Chicago, IL, USA). All tests of significance were two-tailed, and statistical significance was set at P < 0.05.

## Supplementary Information

**Additional file 1: Figure S1. **Plasmid profiles for lentivirus-transformed plasmid, pLV-SARS-CoV-2**-**F1abFabNE-GFP, and envelope plasmid, pCMV3-2019-nCoV-Spike (S1+S2). **Figure S2. **Principle of Lentivirus packaging. **Figure S3. **Optimized detection conditions. **Figure S4. **The SARS-CoV-2 pseudovirus stability with respect to time and temperature.

## Data Availability

All data generated or analyzed during this study are included in the article.
