# Supplementary material for: Immunomolecular assay based on selective virion capture by spike antibody and viral nucleic acid amplification for detecting intact SARS-CoV-2 particles
Source: J Nanobiotechnology. 2022 Sep 5;20:399. doi: 10.1186/s12951-022-01558-8 (PMC9444083; doi:10.1186/s12951-022-01558-8)

**Additional file 1: Figure S1:** Plasmid profiles for lentivirus-transformed plasmid, pLV-SARS-CoV-2**-**F1abFabNE-GFP, and envelope plasmid, pCMV3-2019-nCoV-Spike

(S1+S2).


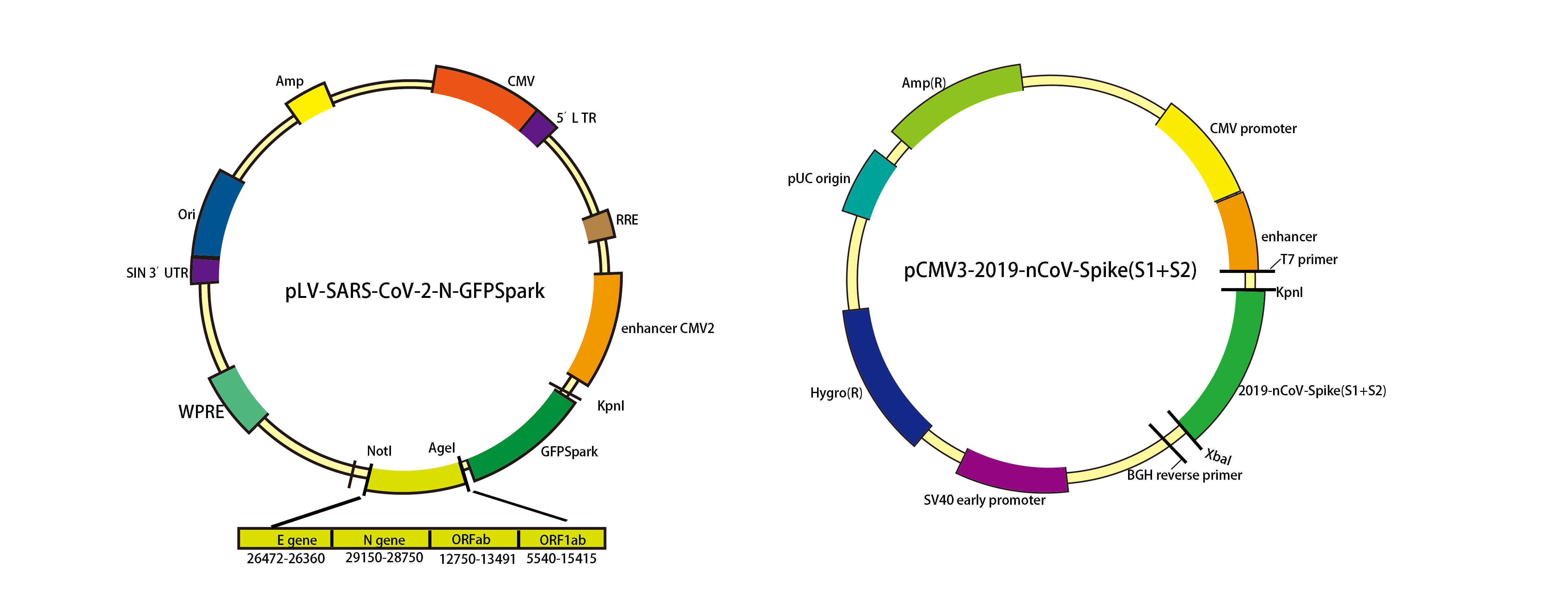


**Additional file 1: Figure S2:** Principle of Lentivirus packaging.


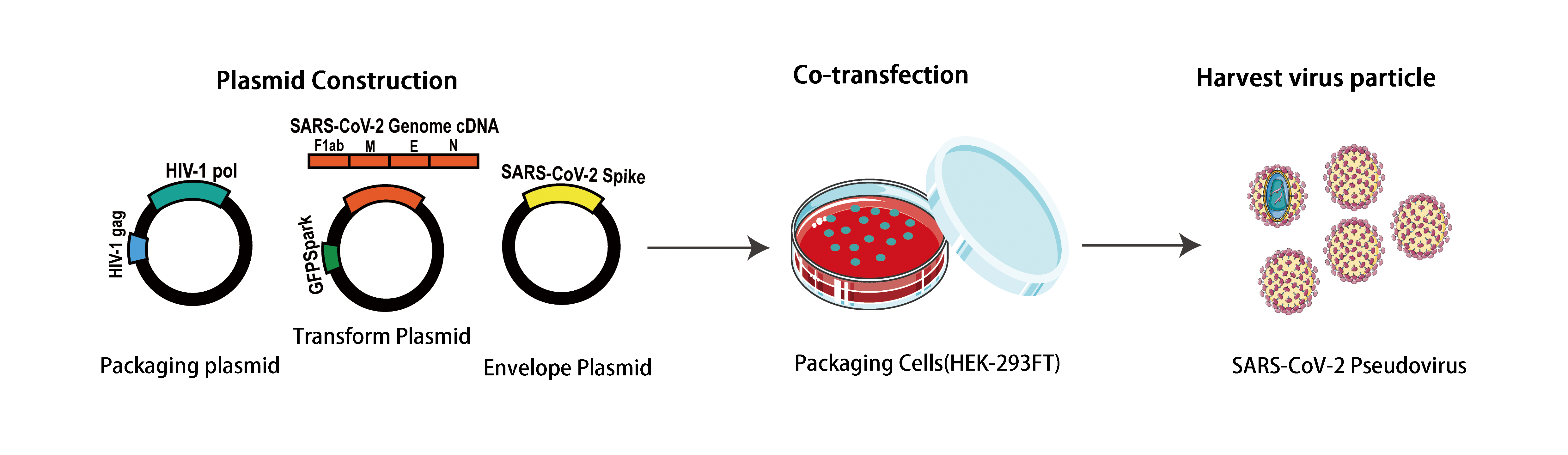


**Additional file 1: Figure S3:** Optimized detection conditions


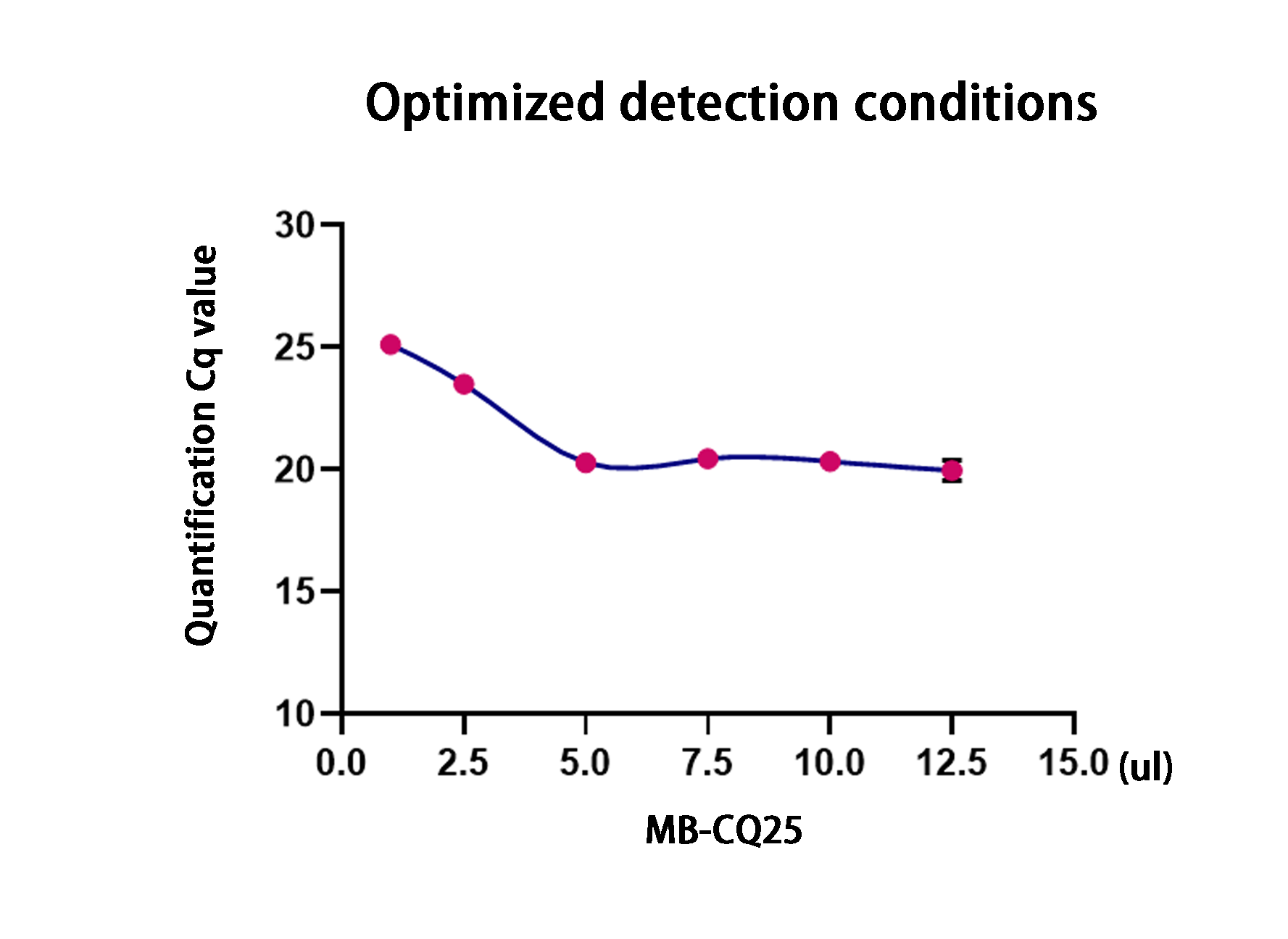


**Additional file 1: Figure S4:** The SARS-CoV-2 pseudovirus stability with respect to time and temperature.


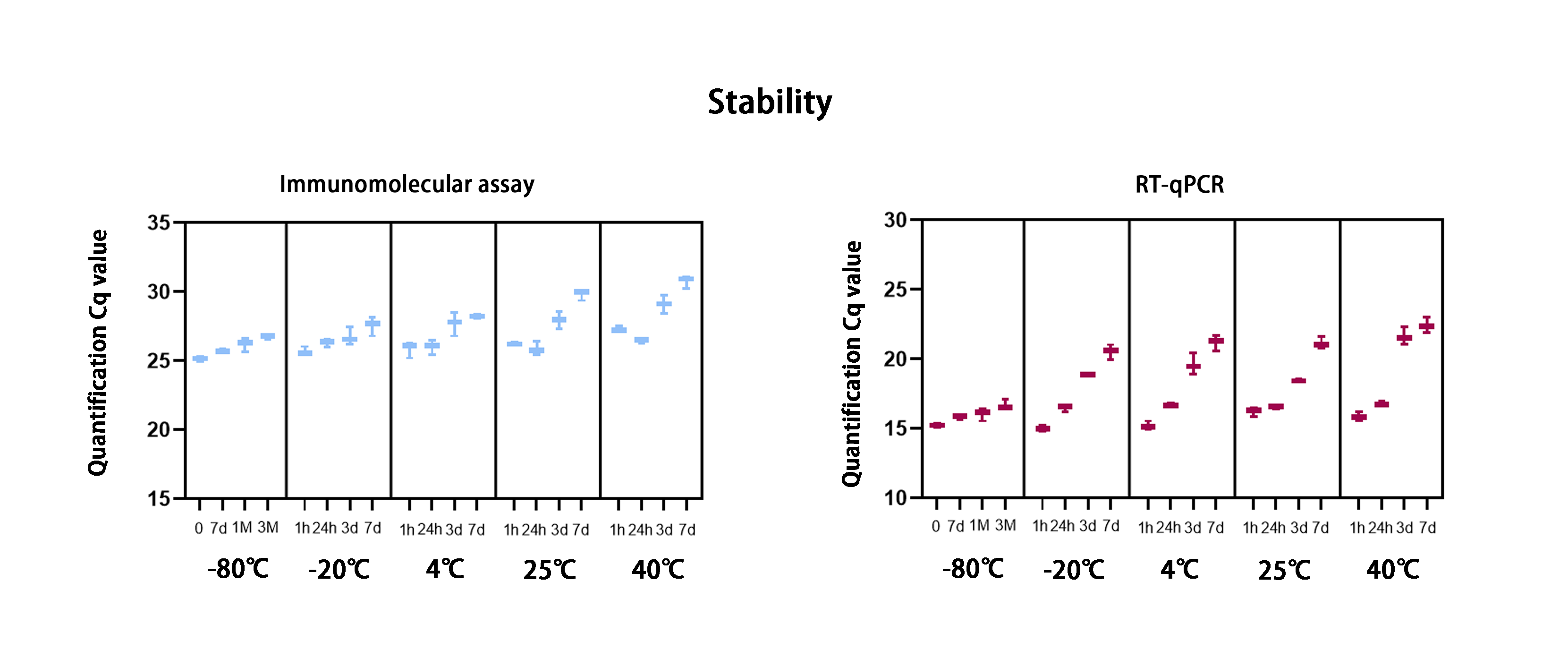

Supplement: Supplementary file 1 — Additional file 1: Figure S1. Plasmid profiles for lentivirus-transformed plasmid, pLV-SARS-CoV-2-F1abFabNE-GFP, and envelope plasmid, pCMV3-2019-nCoV-Spike (S1+S2). Figure S2. Principle of Lentivirus packaging. Figure S3. Optimized detection conditions. Figure S4. The SARS-CoV-2 pseudovirus stability with respect to time and temperature. [file 12951_2022_1558_MOESM1_ESM.docx]
